# Supplementary material for: The interactome of intact mitochondria by cross-linking mass spectrometry provides evidence for coexisting respiratory supercomplexes
Source: Mol Cell Proteomics. 2017 Dec 8;17(2):216–32. doi: 10.1074/mcp.RA117.000470 (PMC5795388; doi:10.1074/mcp.RA117.000470)
Supplement: Supplemental Data [file supp_RA117.000470_133922_0_supp_23978_fzffwf.zip › spectra_annotation/mito_spectra_annotation/278-1-9-1-22-1.pdf]

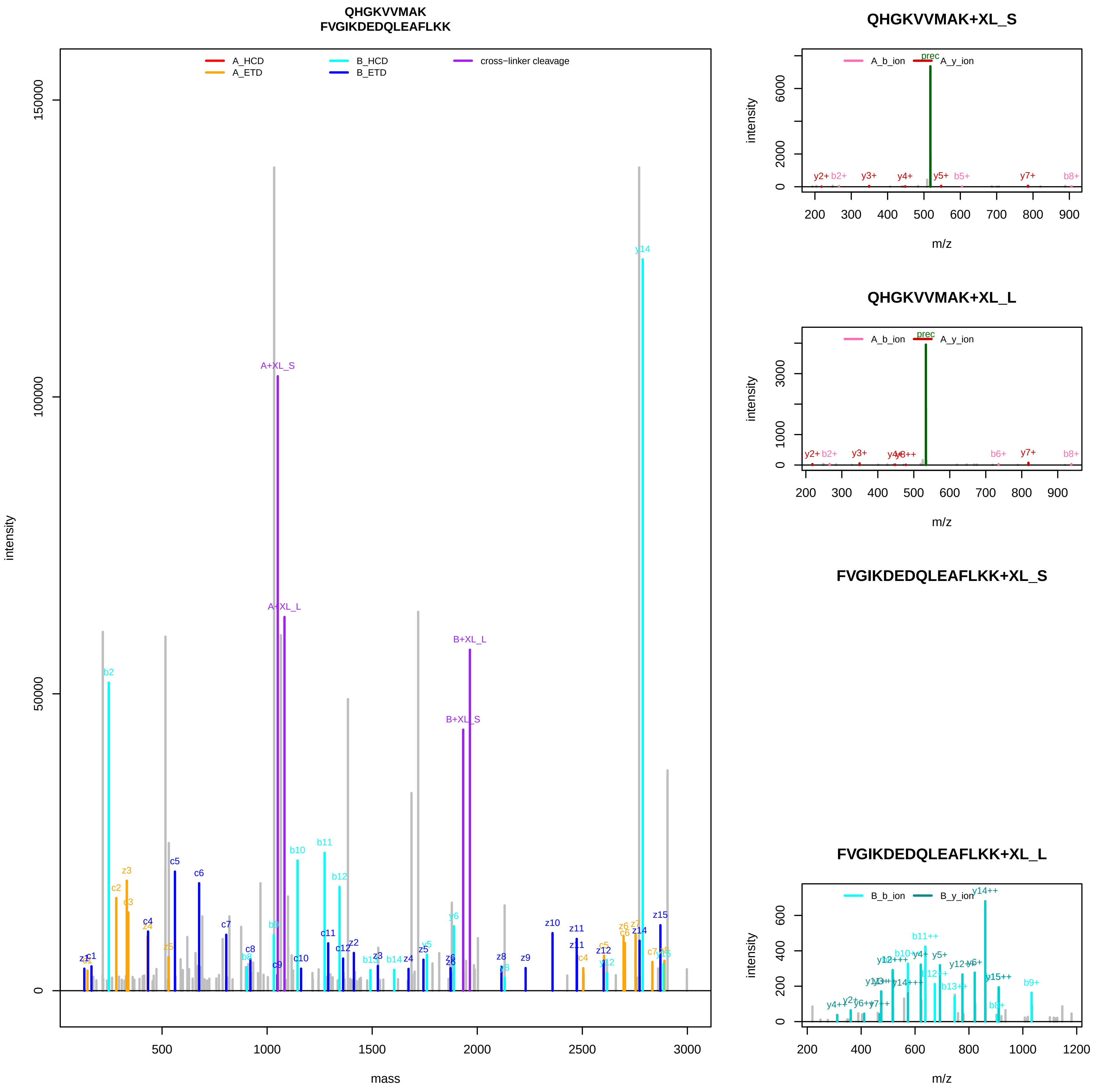

QHGKVVMAK
FVGIKDEDQLEAFLKK
A_HCD
A_ETD
B_HCD
B_ETD
cross−linker cleavage
intensity
mass
0
50000
100000
150000
500
1000
1500
2000
2500
3000
A+XL_S
A+XL_L
B+XL_L
B+XL_S
y14
b2
b10
b11
b12
c5
c6
c7
c8
c9
c10
c11
c12
z3
c2
c3
c4
z4
z5
b8
b9
b13
b14
y5
y6
z8
z9
z10
z11
z12
y12
z14
z15
QHGKVVMAK+XL_S
A_b_ion
A_y_ion
prec
y2+
b2+
y3+
y4+
y5+
b5+
y7+
b8+
m/z
QHGKVVMAK+XL_L
b6+
FVGIKDEDQLEAFLKK+XL_S
FVGIKDEDQLEAFLKK+XL_L
B_b_ion
B_y_ion
y14++
b11++
b10++
y4+
y5+
y12+++
y12++
y6+
y13++
y14+++
b12++
b13++
y15++
b9+
b8+
y4++
y2+
y6++
y7++
